# Molecular Mechanisms Underlying *Vibrio* Tolerance in *Ruditapes philippinarum* Revealed by Comparative Transcriptome Profiling

**DOI:** 10.3389/fimmu.2022.879337

**Published:** 2022-05-09

**Authors:** Zhihui Yin, Hongtao Nie, Kunyin Jiang, Xiwu Yan

**Affiliations:** Engineering and Technology Research Center of Shellfish Breeding in Liaoning Province, College of Fisheries and Life Science, Dalian Ocean University, Dalian, China

**Keywords:** *Ruditapes philippinarum*, *Vibrio anguillarum*, RNA-seq - RNA sequencing, molecular mechanisms, immune response

## Abstract

The clam *Ruditapes philippinarum* is an important species in the marine aquaculture industry in China. However, in recent years, the aquaculture of *R. philippinarum* has been negatively impacted by various bacterial pathogens. In this study, the transcriptome libraries of *R. philippinarum* showing different levels of resistance to challenge with *Vibrio anguillarum* were constructed and RNA-seq was performed using the Illumina sequencing platform. Host immune factors were identified that responded to *V. anguillarum* infection, including C-type lectin domain, glutathione S-transferase 9, lysozyme, methyltransferase FkbM domain, heat shock 70 kDa protein, Ras-like GTP-binding protein RHO, C1q, F-box and BTB/POZ domain protein zf-C2H2. Ten genes were selected and verified by RT-qPCR, and nine of the gene expression results were consistent with those of RNA-seq. The lectin gene in the phagosome pathway was expressed at a significantly higher level after *V. anguillarum* infection, which might indicate the role of lectin in the immune response to *V. anguillarum*. Comparing the results from *R. philippinarum* resistant and nonresistant to *V. anguillarum* increases our understanding of the resistant genes and key pathways related to *Vibrio* challenge in this species. The results obtained here provide a reference for future immunological research focusing on the response of *R. philippinarum* to *V. anguillarum* infection.

## Introduction

*Vibrio anguillarum* is a marine pathogen that can cause fatal hemorrhagic sepsis (vibriosis) in farmed and wild fish, as well as mollusks and crustaceans (1–3). Various studies have focused on immune responses and molecular characteristics of *V. anguillarum* infection in mollusks, such as *Sinonovacula constricta*, *Mytilus galloprovincialis*, and *Chlamys farreri* (4–6). Mollusks lack adaptive immunity and are completely dependent on innate immunity to resist invasion by potentially harmful microorganisms. Work on razor clam *S. constricta* demonstrated the important role of galectins as pattern recognition receptors in innate immunity, with results suggesting that ScGal2 has an indispensable role in the recognition of Gram-negative bacteria in this species (4). Immune responses induced by *V. anguillarum* were investigated in the hepatopancreas of *M. galloprovincialis* using proteomics and metabolomics (5). In *C. farreri*, it was reported that the tumor suppressor QM gene can protect against challenge with pathogens such as *V. anguillarum* (6). Comparative analysis of the immune responses of *Crassostrea gigas* under challenge from different *Vibrio* strains and conditions demonstrated genes could be used as immune responsive biomarkers to monitor early changes in oysters in response to bacterial infection (7).

*Ruditapes philippinarum*, a traditionally commercial clam with high nutritive value and delicate flavor, is widely distributed along the coasts of China, Japan, and Korea (8). It has many advantages as an aquaculture species, including wide salinity and temperature resistance, rapid growth, and pollution tolerance (9). Aquaculture of *R. philippinarum* has faced tremendous challenges caused by bacterial disease, protistan parasites, and environmental stressors (10–12). Vibriosis, a hemorrhagic septicemic disease caused by the bacterium *V. anguillarum*, is an important bacterial infection in Manila clam, which may lead to the death of farmed clams and causing economic losses (13). Several studies using transcriptome sequencing techniques have explored the use of shellfish for disease resistance immunity (14, 15). Transcriptomic analysis of *R. philippinarum* under *V. anguillarum* infection revealed simple sequence repeats and single nucleotide polymorphisms in response to infection (13). More recently, a transcriptome study analyzed the toll-like receptor (TLR) family in *R. philippinarum* after *V. anguillarum* infection to determine the molecular classification and evolutionary model in invertebrates and the basis of the innate immune response of the TLR signaling pathway (16). Transcriptome analyses of responses and defense against different pathogen-associated molecular patterns in *R. philippinarum* were also reported (8). However, the molecular mechanisms underlying different tolerances and levels of resistance to *V. anguillarum* in *R. philippinarum* have yet to be fully elucidated. Studying the immune molecular mechanism of clams in different states is helpful for analysis of fundamental differences between the susceptible and resistant groups. Selecting resistant individuals in clam production for breeding is in turn beneficial for improving production capacity and economic benefits. Hepatopancreas is an important immune-related tissue and an organ for biotransformation and detoxification of exogenous organisms (13, 17), especially under the stimulation of lipopolysaccharide (LPS), the involvement of immune response-related genes in hepatopancreatic tissue was also significantly higher than that in other tissues (18). In the present study, *R. philippinarum* was challenged with *V. anguillarum* and transcriptome sequencing was performed on individuals resistant and susceptible to infection. The study provides insights into the molecular basis and potential regulatory mechanisms underlying vibrio tolerance-related factors in *R. philippinarum* under *V. anguillarum* infection.

## Materials and Methods

### *R. philippinarum* and *V. anguillarum* Challenge

**Figure 1:**
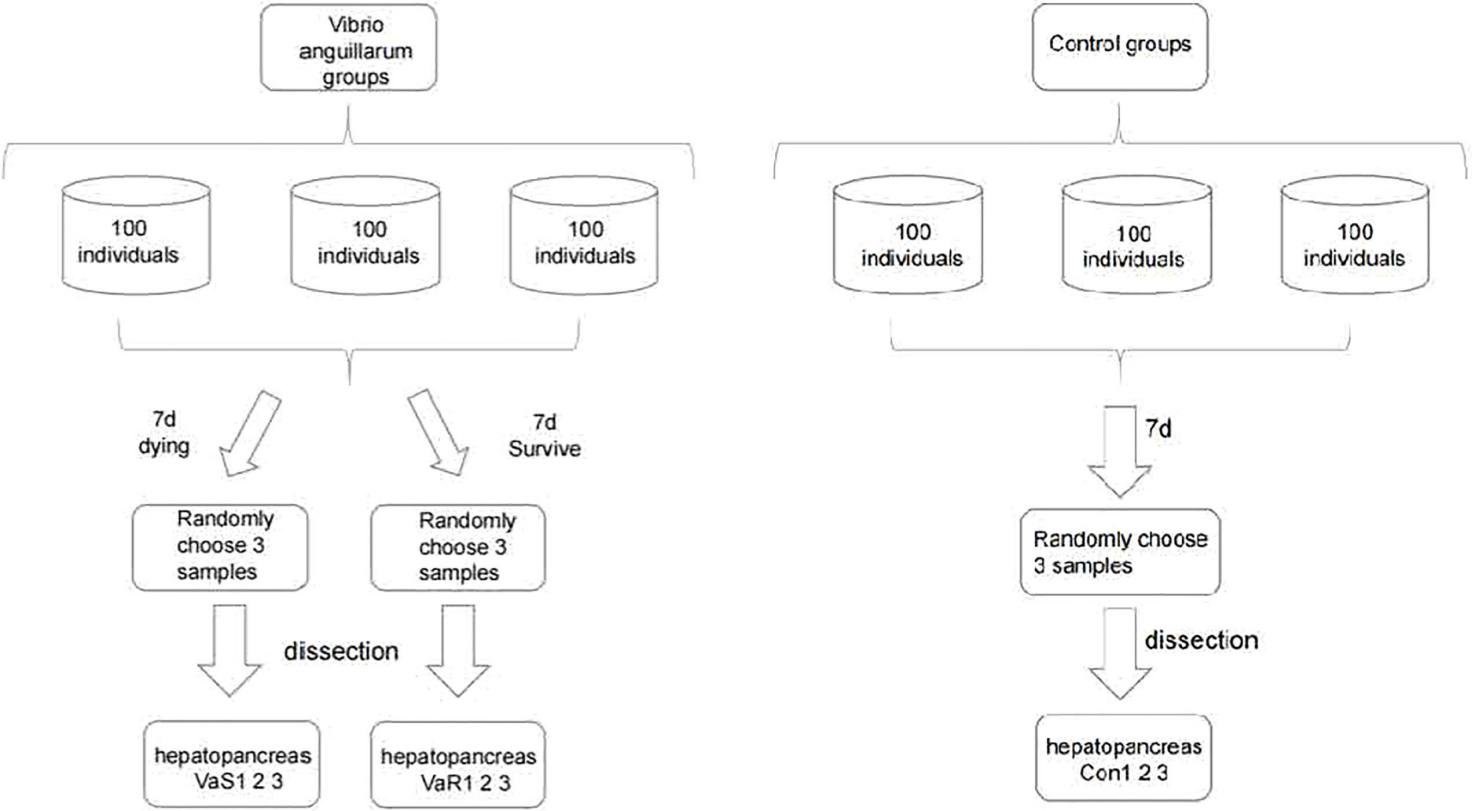
The experimental design and flow chart of sampling in each group of *Vibrio anguillarum* stress (VaS, VaR) and control group (Con). Manila clam was sampled from each group on the seventh day after *V. anguillarum* challenge. In order to ensure the randomness of sampling, three clams were randomly collected in each group as three biological replicates.

The *R. philippinarum* used in this study were a wild population collected from the coast of Jinzhou, Dalian, Liaoning Province, China. The clams were cleaned to remove any fouling and were acclimated in 50-L aerated plastic tanks (water temperature: 21 ± 0.3°C, pH 8.2 ± 0.1, salinity 32 ± 0.2 ppt). Chlorella powder was feed daily during the two weeks acclimatation period, and the status of normal surviving clams was observed every day, and some clams were randomly selected for dissection to observe whether the state of each tissue was normal. The selected experimental material is determined to be healthy wild clams without *V. anguillarum* infection. After the clams acclimated to the laboratory environment, stop feeding and start the experiment. The clams had an average shell length of 34.8 ± 0.4 mm, an average width of 16.4 ± 0.2 mm, an average height of 24.1 ± 0.3 mm, and an average weight of 8.4 ± 0.4 g. The concentration of *V. anguillarum* used in the study was 10^7^ colony-forming units (CFU)/mL. The *R. philippinarum* were divided at random into an *V. anguillarum* stress group and an untreated control group (each group *n* = 100). **Figure 1** shows the experimental setup and details of the procedures for sample collection. The number of death clams was counted every 24 h, and the cumulative mortality of the *V. anguillarum* stress group and the control group were calculated respectively. The calculation method of the cumulative mortality is: cumulative mortality equals the ratio of the number of deaths to the total number of each group. According to the 16-day number of deaths statistical data, the highest mortality occurred on Day 7 of the experiment. Therefore, the clams were sampled and sequenced on Day 7 of the experiment. The sample tissue selection method included several steps. One susceptible individual per group was selected from the three parallel groups after the *V. anguillarum* group. A total of three individuals were selected for the susceptible group (VaS; i.e., adductor muscle was not closed, dying state/moribund). Then, one susceptible individual per group was selected from the three parallel groups after the *V. anguillarum* group. A total of three individuals were selected for the resistant group (VaR; normal survival). One individual from each of the three parallel groups in the control group was selected for a total of three individuals for the control group (Con). The selected nine individuals were dissected and the hepatopancreatic tissues were cut out. The samples were immediately frozen in liquid nitrogen and stored at −80°C before use.

### RNA Extraction and Library Construction for Illumina Sequencing

Use RNAprep pure Tissue Kit (TianGene, Beijing, China) extraction method to extract RNA from hepatopancreas tissue, and then strictly control the quality of the RNA sample. The quality control method is mainly through the Agilent 2100 bioanalyzer, then the RNA integrity is tested, and the experiment is as follows The NEB common library building method is used to build the library (19), using fragmented mRNA as a template, random oligonucleotides as primers, and synthesizing the first strand of cDNA in the M-MuLV reverse transcriptase system Second strand cDNA synthesis was subsequently performed using DNA Polymerase I and RNase H. After adenylation of 3′ ends of DNA fragments, NEBNext Adaptor with hairpin loop structure was ligated to prepare for hybridization (20). To select cDNA fragments of preferentially 250 ~ 300 bp in length, the library fragments were purified with AMPure XP system (Beckman Coulter, Beverly, USA) Then 3 μL USER enzyme (NEB, USA) was used with sizeselected, adaptor-ligated cDNA at 37°C for 15 min followed by 5 min at 95°C before PCR. PCR was performed with Phusion High-Fidelity DNA polymerase, Universal PCR primers, and Index (X) Primer. At last, PCR products were purified (AMPure XP system) and library quality was assessed on the Agilent Bioanalyzer 2100 system (8, 21).

### Sequence Filtering, Mapping and Assembly

Since the raw data obtained by sequencing contains a small number of reads with sequencing adapters and low sequencing quality, in order to ensure the quality and reliability of data analysis, we filter the raw data to remove reads with adapters; remove the reads containing N (N means that the base information cannot be determined) reads; remove low-quality reads (reads whose base number of Qphred<=20 accounts for more than 50% of the entire read length). The raw sequences were deposited in the National Center for Biotechnology Information (NCBI) Short Read Archive (SRA) database under accession number PRJNA738278 (http://www.ncbi.nlm.nih.gov/Traces/sra/). Feature Counts v1. 5. 0-p3 was used to count the reads numbers mapped to each gene, and then FPKM of each gene was calculated based on the length of the gene and reads count mapped to this gene (8, 22). StringTie assembles the genes for each data set separately, estimating the expression levels of each gene and each isoform as it assembles them (8). StringTie uses network streaming algorithms and optional *de novo* assembly to splice transcripts (23).

### Differential Expression Analysis

After the gene expression quantification is completed, we perform statistical analysis on the expression data, and screen the samples with significantly different expression levels in different states. Differential expression analysis was performed using the DESeq2 R package (24). DESeq2 provides statistical routines for determining differential expression in digital gene expression data using a model based on the negative binomial distribution (24). The experiment divides the difference analysis into three main steps. First, the original readcount is normalized, mainly to correct the sequencing depth. Then the statistical model performs the calculation of the hypothesis test probability (P-value), The resulting P-value were adjusted using the Benjamini and Hochberg’s approach for controlling the false discovery rate (25). Genes with an padj < 0.05 founded by DESeq2 were assigned as differentially expressed (8). and finally performs the multiple hypothesis test correction to obtain the FDR value (26–28). Screening of differentially expressed genes (DEGs), which are the core basis of transcriptome sequencing analysis, are genes with large differences in expression in different comparison combinations, or shared differential genes, which can be used as key genes.

### Enrichment Analysis of GO and KEGG Differentially Expressed Genes

The experiment uses clusterProfile software to perform GO function enrichment analysis on the differential gene set. When the padj is less than 0.05, it is considered that the differentially expressed genes (DEGs) is significantly enriched in GO (24). We use clusterProfile software to perform KEGG pathway enrichment on the differential gene set analyze. KEGG (Kyoto Encyclopedia of Genes and Genomes) is a comprehensive database that integrates genome, chemistry, and system function information. KEGG pathway enrichment takes padj less than 0.05 as the threshold of significant enrichment.

### qPCR Confirmation of Illumina Sequencing Data

To validate the Illumina sequencing data, ten immune-related DEGs were chosen for quantitative realtime PCR (qPCR) analysis. The specific primers for these genes are shown in **Table S1**. The integrity and purity of RNA were electrophoresed using 1% agarose gel and NanoDrop ND-2000 spectrophotometer (Thermo Electron Corp, Waltham, Massachusetts, USA), respectively. The total RNA was reverse transcribed into cDNA using PrimeScript RT kit (Takara, Tokyo, Japan). Primer 5 software (Premier Biosoft International) was used to design primers. β-actin was selected as the reference gene for qPCR analysis due to its stable expression characteristics (18, 29). Quantitative PCR used TB Green PreMix ExTaqII (Takara, Tokyo, Japan). The total volume of the reaction is 20 μL, which contains 2 μL of diluted cDNA (50 μg/μL), 1 μL of each primer, 10 μL of TB Green PCR Master Mix and 6 μL of H2O. The cycle curve is as follows: 94°C 5min, 94°C 40 Cycle for 30s, 60°C for 30s, 72°C for 30s, and each sample was processed into three copies in the Roche LightCycler480 Real-time PCR system (Roche LightCycler480 Real-time PCR system, Roche LightCycler480 Real-time PCR system). The expression level was analyzed using the 2^-ΔΔCT^ method (30). Significant differences among sample data were analyzed using SPSS 20.0. We conducted a One-Way analysis of variance (ANOVA) with the Tukey test to compare the significant differences Survival status groups. Differences were considered significant at *P* < 0.05 (31).

## Results

### Cumulative Mortality Results

**Figure 2:**
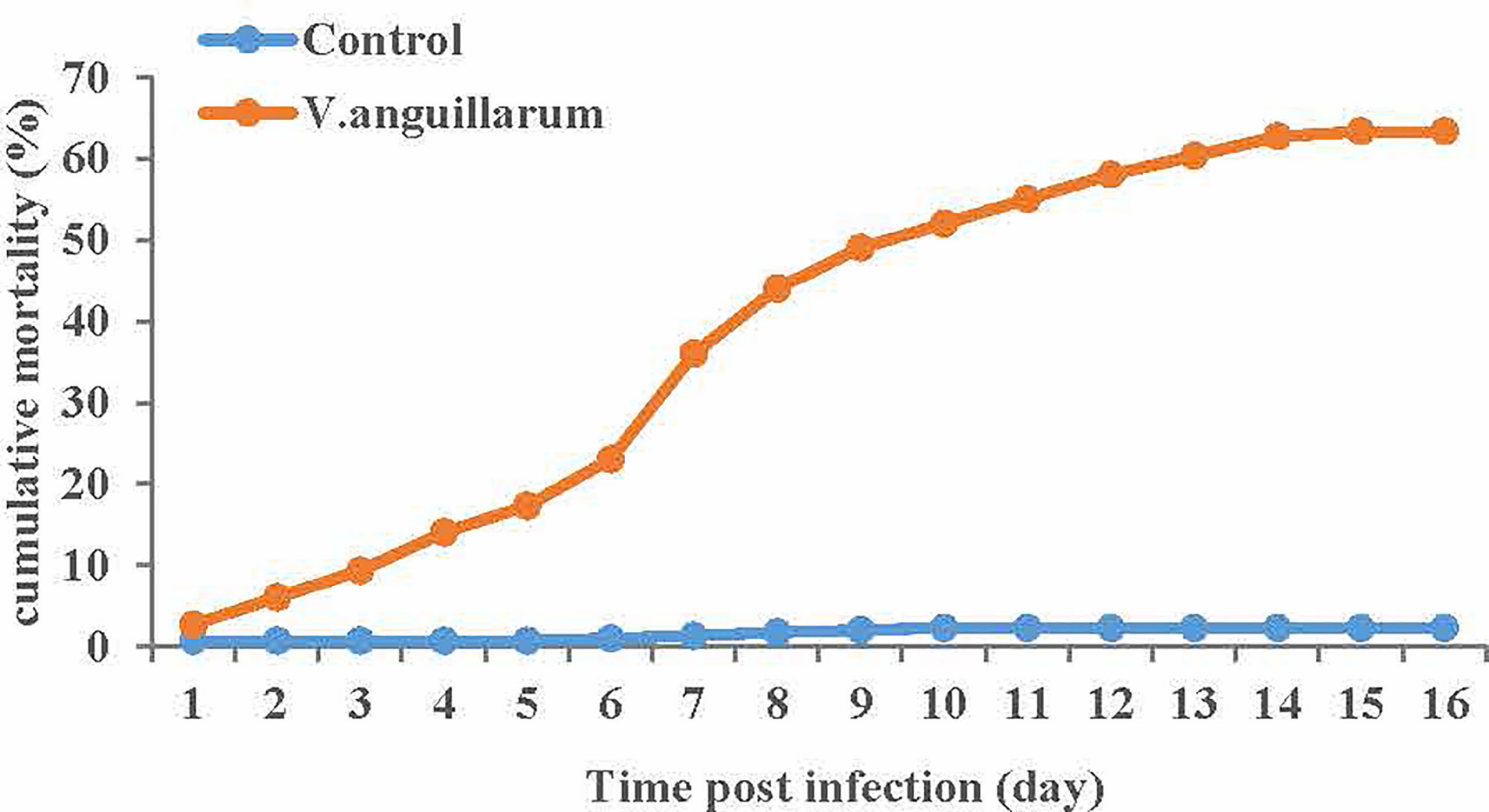
Average cumulative mortality statistics of each of the three replicates of the *V. anguillarum* challenge group and the control group. Broken-line statistical graph of mortality of the experimental group and the control group, the abscissa is the number of days in the experiment, the ordinate is the cumulative mortality. The cumulative mortality calculation method is the ratio of the cumulative number of deaths to the starting number. Red represents the *V. anguillarum* stress group, blue represents the control group, and each node represents the cumulative mortality value of the corresponding group on the day.

The cumulative mortality and deaths number of clams in response to *V. anguillarum* infection was recorded every 24 h for 16 days. We plotted the cumulative mortality **Figure 2** of the *V. anguillarum* stress group and the control group, as show in (**Table S2**). the *V. anguillarum* stress group had the highest number of deaths on the seventh day of the experiment. During the whole experiment, on the 16th day, the average cumulative mortality of the three parallel groups was 63.3% in the *V. anguillarum* stress group, and the average cumulative mortality in the control group was less than 2.3%.

### Genome-Guided Transcriptome Assembly

**Figure 3:**
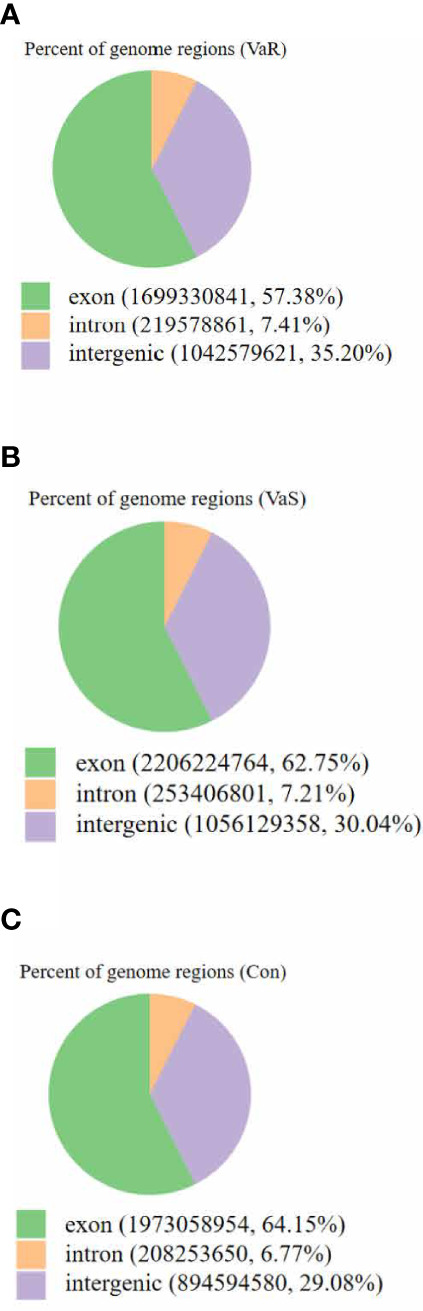
The distribution of sequencing reads in the genomic region. The different color ratios in the figure represent the ratio of reads compared to different areas. Exon: The number of reads aligned to the exon region of the genome and its ratio to the number of clean reads. Intron: The number of reads compared to the intron region of the genome and its proportion to the number of clean reads. Intergenic: The number of reads compared to the intergenic region and its ratio to the number of clean reads. **(A)** VaR (resistant group), **(B)** VaS (susceptible group), **(C)** Con (control group).

In total, 135 427 218 raw reads were obtained, including 44 869 722 raw reads from the VaS group, 44 827 447 from the VaR group, and 45 730 048 from the Con group (**Table 1**). After excluding low-quality reads (mass fraction<20) (8), short reads (length<60 bp) and unclear nucleotides, 44 049 293 clean reads were reserved for further mapping and differential expression analysis. The proportion of reads in the exon, intron, and intergenic regions of the genome in each group was calculated (**Figure 3**).

**Table 1:** After filtering the original data, checking the sequencing error rate, and checking the GC content distribution, we obtain clean reads for subsequent analysis. The data is summarized as shown in the table below.

Sample	Library	Raw_reads	clean_reads	Clean_bases	Error_rate	Q20	Q30	GC_pct
tVaS1	FRAS202156226-1r	43614846	42016796	6.3G	0.03	97.94	93.92	37.1
tVaS2	FRAS202156227-1r	45933166	44134864	6.62G	0.03	97.58	93.11	37.7
tVaS3	FRAS202156228-1r	45061154	43428666	6.51G	0.02	98.07	94.26	37.71
tVaR1	FRAS202156223-1r	43504158	42181696	6.33G	0.03	97.97	93.96	35.83
tVaR2	FRAS202156224-1r	44238968	42270438	6.34G	0.03	97.72	93.37	37.54
tVaR3	FRAS202156225-1r	46739216	43630738	6.54G	0.03	97.79	93.57	37.48
tCon1	FRAS202156229-1r	44446884	42744414	6.41G	0.02	98.04	94.16	38.45
tCon2	FRAS202156230-1r	45099750	43562032	6.53G	0.03	97.95	94	37.95
tCon3	FRAS202156231-1r	47643512	45841434	6.88G	0.03	98.01	94.12	38.14

Sample: Sample name.

Library: Library number.

Raw_reads: The number of reads in the raw data.

Clean_reads: The number of reads after filtering the original data.

Clean_bases: The number of bases after filtering the original data (clean base=clean reads*150bp).

Error_rate: The overall sequencing error rate of the data.

Q20: The percentage of bases with a Phred value greater than 20 to the total bases.

Q30: The percentage of bases with a Phred value greater than 30 to the total bases.

GC_pct: the percentage of G and C in the four bases in clean reads.

### Detection of Differentially Expressed Genes

**Figure 4:**
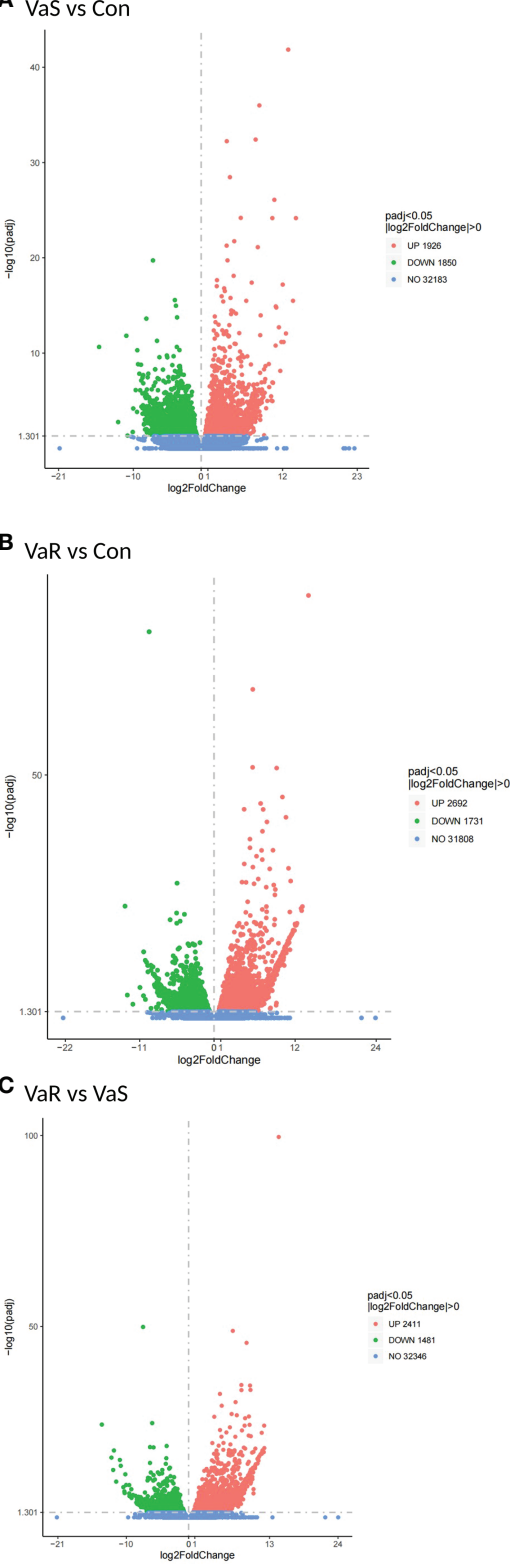
Differential gene volcano map. **(A)** VaR vs. Con; **(B)** VaR vs. VaS; **(C)** VaS vs. Con; x-axis, expression ratio of the different samples; y-axis, significance of the differential gene expression, which is positive in relation to the –log10 (padj) value. Red plots represent the upregulated genes; green plots represent the downregulated genes; and blue plots represent no significant difference. UP, upregulated genes; DOWN, downregulated genes; NO, no significant difference. VaR (resistant group), VaS (susceptible group), Con (control group).

**Figure 5:**
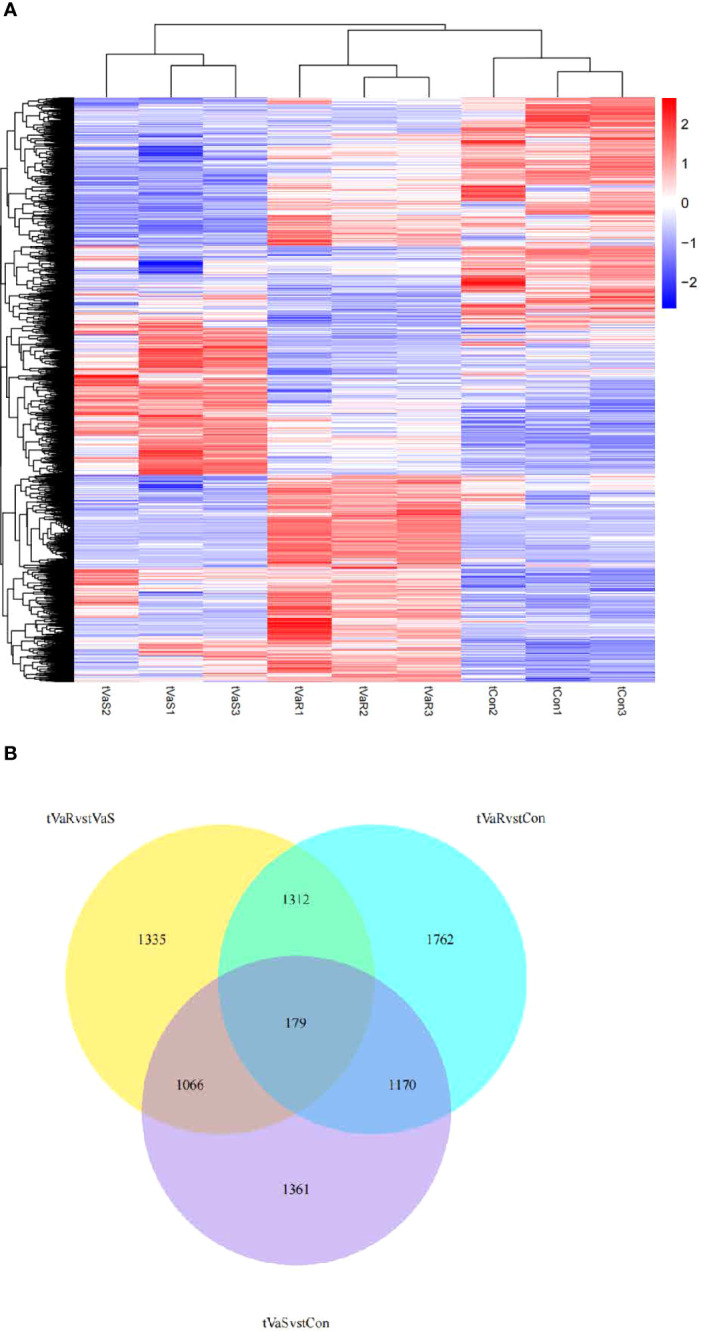
**(A)** Differentially expressed gene clustering heat map. In the figure, the abscissa is the sample name, and the ordinate is the normalized value of the differential gene FPKM. The stronger the red color, the higher the expression level, and the greener, the lower the expression level. **(B)** Differential Gene Venn Diagram. Different colors indicate different comparison combinations.

Compared with the control group, 3776 differentially expressed genes (DEGs) were identified in the *V. anguillarum* challenge group (VaS vs Con), including 1926 upregulated genes and 1850 downregulated genes (**Figure 4A**). A total of 4423 DEGs were identified in the VaR group compared with the Con group, including 2692 upregulated genes and 1731 downregulated genes (**Figure 4B**). There were 2411 upregulated genes and 1481 downregulated genes in the VaR group compared with the VaS group (**Figure 4C**). There were significantly more upregulated genes in the VaR vs Con group than in the VaS group (**Figure 4**). To reveal the activated immune response genes in the different groups, we further analyzed the transcripts based on the critical value (≥2 times change, *P*<0.05). We performed a hierarchical cluster map of the genes, revealing the overall expression profile of DEGs in each group (**Figure 5**), suggesting that the VaR group first clustered with the control group, and then with the VaS group. We also used Venn diagrams to show the overlap of DEGs between different comparison combinations (**Figure 5**), and screened out 179 DEGs and unique DEGs from the three comparison combinations. We verified and described pattern recognition receptors (PRRs), such as C1q and C-type lectin, and genes related to immune stress, such as heat shock protein (HSP) and lysozyme gene. The detailed DEG results are shown in (**Table 2**).

**Table 2:** Differential genes included in each comparative combination after *Vibrio anguillarum* stress.

Abbreviations	Gene name	ID	Log2 Fold change		
			VaR vs. Con	VaR vs. VaS	VaS vs. Con
fn3	Fibronectin type III domain	evm.TU.xfSc0001199.7	8.333	3.122	5.186
Guanylate_cyc	Atrial natriuretic peptide receptor 2	evm.TU.xfSc0001277.8	7.515	3.460	4.035
Lipase	Pancreatic triacylglycerol lipase	evm.TU.xfSc0000254.4	7.085	-2.376	9.442
Ras	Ras-like GTP-binding protein RHO	evm.TU.xfSc0001526.12	6.017	-6.514	12.495
VWA	von Willebrand factor type A domain	evm.TU.xfSc0000414.8	5.713	-2.333	8.027
Lipase	Inactive pancreatic lipase-related protein 1	evm.TU.xfSc0000254.3	5.176	-3.541	8.704
TSP_1	Transmembrane protein	evm.TU.xfSc0001422.5	4.786	-4.185	8.931
ubiquitin	Polyubiquitin-C	evm.TU.xfSc0000211.11	4.605	-6.207	10.789
ubiquitin	Ubiquitin family	evm.TU.xfSc0001916.3	4.249	-2.827	7.059
PG_binding_1	Matrix metalloproteinase-14	evm.TU.xfSc0000153.14	3.645	-1.504	5.134
BIR	Baculoviral IAP repeat-containing protein	evm.TU.xfSc0001914.6	3.115	-1.786	4.876
EGF_CA	Dorsal-ventral patterning protein	evm.TU.xfSc0000498.10	2.686	-1.772	4.444
VWA	Collagen alpha-5(VI) chain	evm.TU.xfSc0000204.3	2.643	4.699	-2.081
Lectin_C	Lectin C-type domain	evm.TU.xfSc0000050.5	2.596	-1.673	4.264
GTP_EFTU	Elongation factor 2	evm.TU.xfSc0000001.41	2.514	-1.273	3.756
TIR	Toll-like receptor	evm.TU.xfSc0000165.21	2.397	-1.406	3.775
SAM_1	SAM domain (Sterile alpha motif)	evm.TU.xfSc0002113.3	-3.891	4.080	-7.994
Peptidase_S8	Proprotein convertase subtilisin/kexin type	evm.TU.xfSc0002219.4	-5.590	-2.649	-2.970

### GO and KEGG Enrichment Analysis DEGs

**Figure 6:**
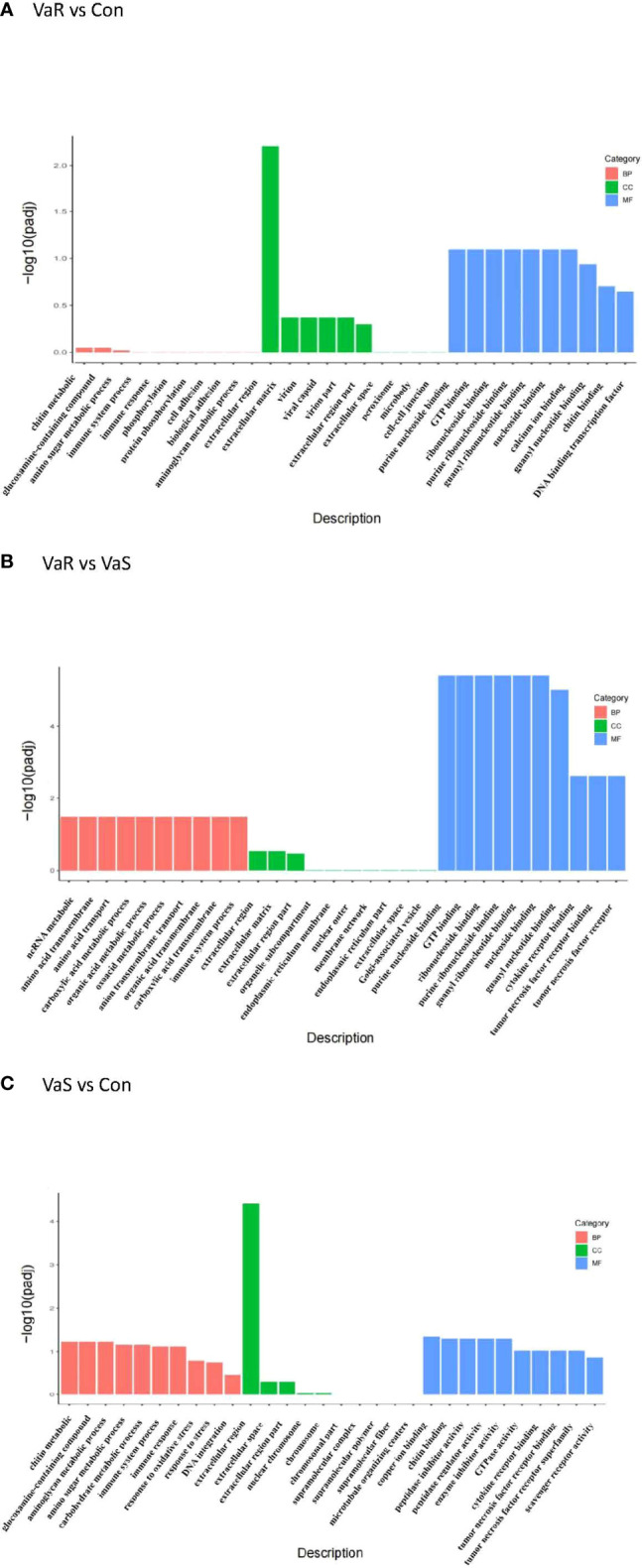
GO enrichment analysis histogram. From the GO enrichment analysis results, select the most significant 30 terms to draw a histogram for display. If there are less than 30 terms, draw all terms, as shown in the figure below. In the figure, the abscissa is GO Term, and the ordinate is the significance level of GO Term enrichment. which is represented by -log10 (padj). The higher the value, the more significant. Different colors represent the three GO sub-categories BP, CC, and MF. **(A)** VaR vs Con, **(B)** VaR vs VaS, **(C)** VaS vs Con. VaR (resistant group), VaS (susceptible group), Con (control group).

The DEGs from each group were analyzed by GO enrichment analysis. In each comparison combination, the abscissa was the GO Term, and the ordinate was the significant level of GO Term enrichment (**Figure 6**). The higher the value, the higher its significance. Different colors in **Figure 6** represent the GO subclasses of biological processes, cellular components, and molecular functions. The top GO terms (i.e. those with the highest enrichment) were mainly related to extracellular regions (GO:0005576), copper ion binding (GO:0005507), chitin binding (GO:0008061), peptidase inhibitor activity (GO:0030414), peptidase regulator activity (GO:0061134), enzyme inhibitor activity (GO:0004857), non-coding RNA metabolic processes (GO:0034660), amino acid transmembrane transport (GO:0003333), amino acid transport (GO:0006865), and carboxylic acid metabolic processes (GO:0019752).

**Figure 7:**
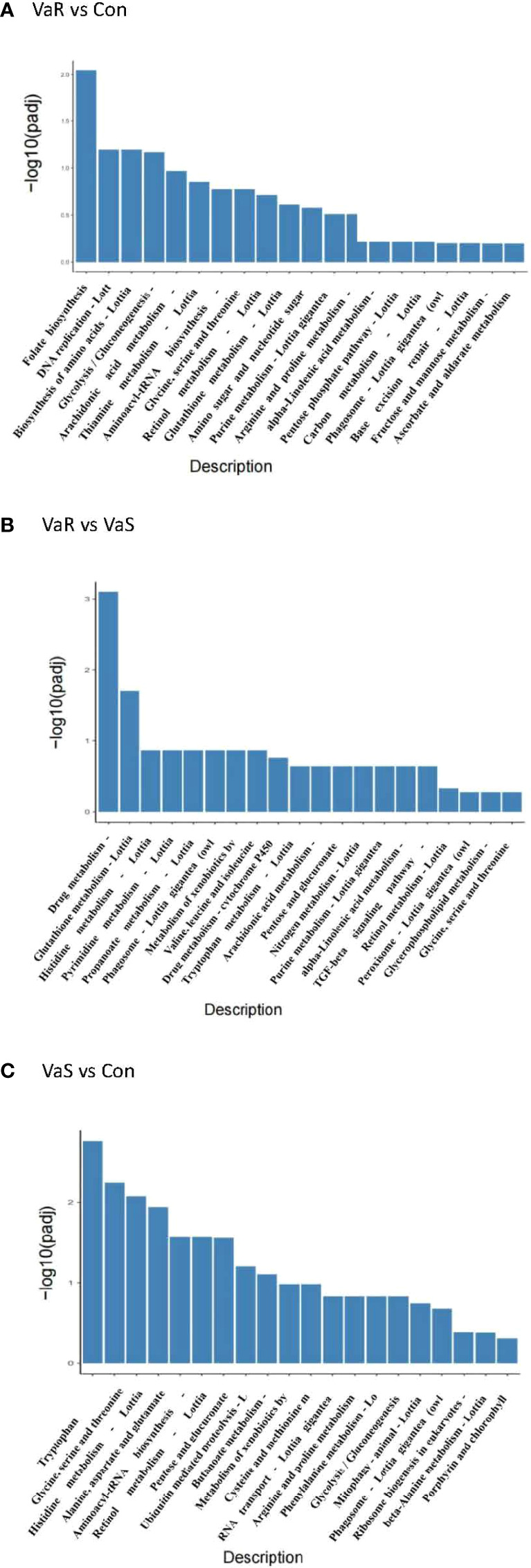
KEGG enrichment analysis histogram. The abscissa in the figure is the KEGG pathway, and the ordinate is the significance level of pathway enrichment. The higher the value, the more significant it is **(A)** VaR vs Con, **(B)** VaR vs VaS, **(C)** VaS vs Con. VaR (resistant group), VaS (susceptible group), Con (control group).

The KEGG pathway enrichment analysis of DEGs is shown in **Table 3**. From these results, the pathways that showed the most significant changes were detected (**Figure 7**). There was a significantly enriched pathway in the VaR group compared with the Con group folate biosynthesis-*Lottia gigantea* (lgi00790). Compared with the VaS group, the VaR group had two significantly enriched pathways: drug metabolism-other enzymes-*L. gigantea* (lgi00983) and glutathione metabolism-*L. gigantea* (lgi00480), whereas there were more pathways that were significantly enriched between the VaS versus Con groups: tryptophan metabolism-*L. gigantea* (lgi00380); glycine, serine, and threonine metabolism-*L. gigantea* (lgi00260); histidine metabolism-*L. gigantea* (lgi00340); alanine, aspartate, and glutamate metabolism-*L. gigantea* (lgi00250); aminoacyl-tRNA biosynthesis-*L. gigantea* (lgi00970); retinol metabolism-*L. gigantea* (lgi00830); pentose and glucuronate interconversions-*L. gigantea* (lgi00040); and phagosome (lgi04145).

**Table 3:** ClusterProfile software was used to perform KEGG pathway enrichment analysis on the differential gene set.

KEGGID	Description	DGEs number	pvalue
lgi00790	Folate biosynthesis	10	0.000
lgi03030	DNA replication	10	0.002
lgi01230	Biosynthesis of amino acids	16	0.002
lgi00010	Glycolysis/Gluconeogenesis	13	0.003
lgi00590	Arachidonic acid metabolism	17	0.005
lgi00730	Thiamine metabolism	7	0.008
lgi00970	Aminoacyl-tRNA biosynthesis	11	0.011
lgi00260	Glycine, serine and threonine metabolism	12	0.012
lgi00830	Retinol metabolism	14	0.016
lgi00480	Glutathione metabolism	17	0.023
lgi00520	Amino sugar and nucleotide sugar metabolism	15	0.027
lgi00230	Purine metabolism	20	0.037
lgi00330	Arginine and proline metabolism	11	0.037
lgi00592	alpha-Linolenic acid metabolism	6	0.048
lgi00030	Pentose phosphate pathway	6	0.064
lgi01200	Carbon metabolism	19	0.067
lgi04145	Phagosome	19	0.105
lgi03410	Base excision repair	6	0.105
lgi00051	Fructose and mannose metabolism	6	0.117
lgi00053	Ascorbate and aldarate metabolism	3	0.124
lgi00340	Histidine metabolism	6	0.129
lgi01040	Biosynthesis of unsaturated fatty acids	6	0.129
lgi03420	Nucleotide excision repair	7	0.140
lgi00900	Terpenoid backbone biosynthesis	4	0.158
lgi00360	Phenylalanine metabolism	3	0.172
lgi00860	Porphyrin and chlorophyll metabolism	4	0.177
lgi04122	Sulfur relay system	4	0.177
lgi00500	Starch and sucrose metabolism	7	0.191
lgi00561	Glycerolipid metabolism	8	0.195

KEGGID: KEGG channel number.

Description: The function description corresponding to the KEGG channel number.

pvalue: Significance test p value.

DGEs number: The number of differential genes annotated to the KEGG pathway number.

### qPCR Validation of Gene Expression Profiles

**Figure 8:**
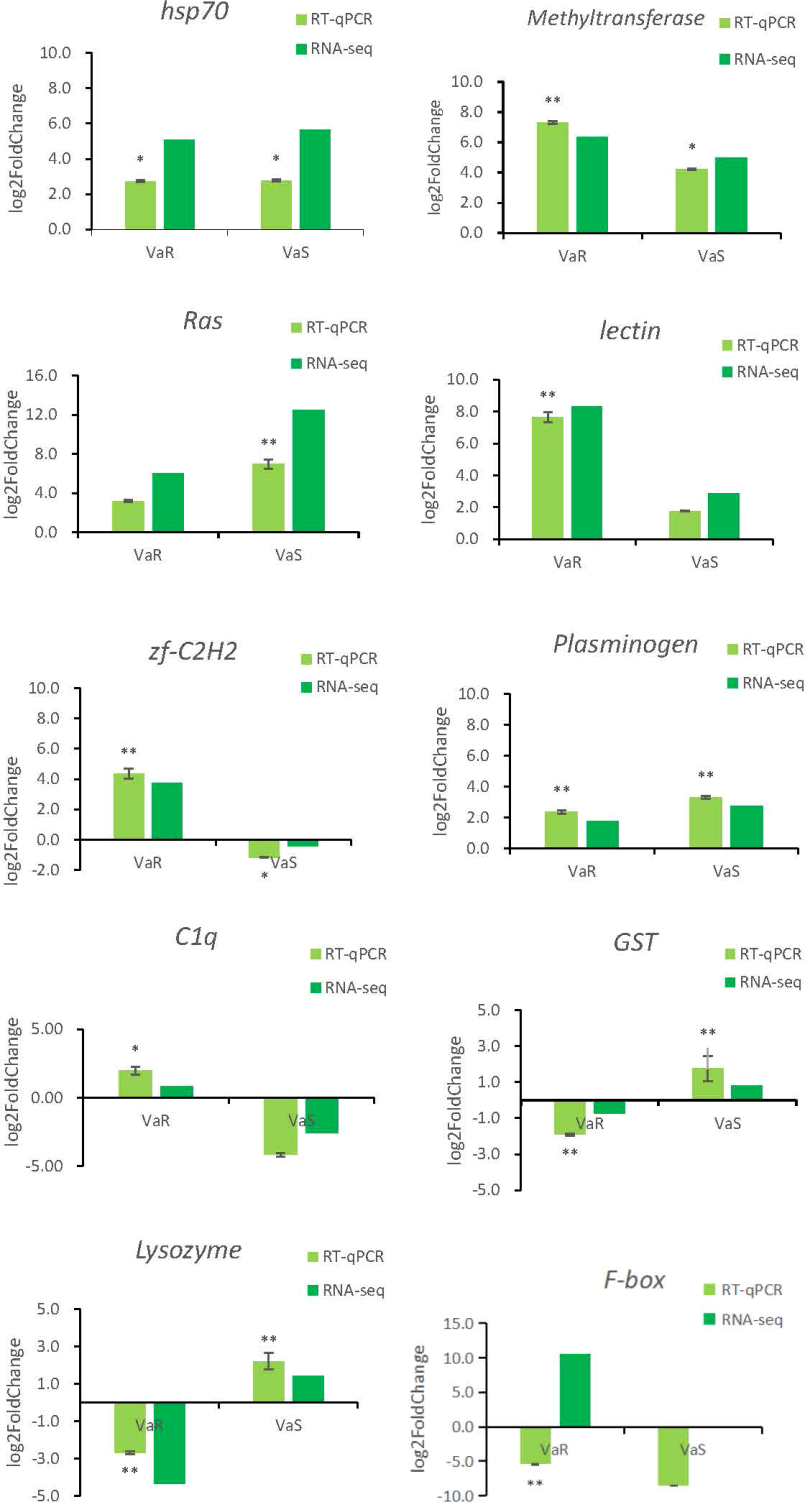
Validation of relative expression levels of ten transcripts by qRT-PCR compared with RNA-seq. The x-axis is the resistance group (VaR) and the susceptible group (VaS) compared with the control group, and the y-axis is the expression of fold change. * Indicates the significance of the fold change difference of VaR and VaS compared with the control group, respectively. * indicates significant (*P* < 0.05), ** indicates extremely significant (*P* < 0.01).

To verify the accuracy of the RNA-sequencing (RNA-seq) results, shared and specific immune-related genes differentially expressed in the Con, VaS, and VaR groups were selected for real-time quantitative PCR (RT-qPCR). The relative expression levels of PRRs, such as C1q and C-type lectin genes, in the VaR group were higher than those in the Con or VaS groups. The C1q and zf-C2H2 gene were remarkably up-regulated in the resistant group and down-regulated in the susceptible group. While the GST and Lysozyme gene were lowly expressed in the resistant group and highly expressed in the susceptible group. In addition, the Methyltransferase, Ras, lectin, and plasminogen were highly expressed in both resistant and susceptible groups, but the expression levels are significantly different. The expression of HSP70 gene were significantly up-regulated in the resistant group and the susceptible group with a similar trend. The fold change detected by qPCR was compared with that detected by RNA-Seq expression analysis (**Figure 8**). In general, the genes identified by RT-qPCR were consistent with the results of Illumina sequencing analysis, indicating the accuracy of the RNA-seq expression analysis.

## Discussion

Research has suggested that evolution of *Rp*TLRs is associated with their immune recognition and function in *R. philippinarum* in response to *V. anguillarum* (16). Transcriptome analyses of the immune response of *R. philippinarum* to infection with *V. anguillarum* identified many candidate immune-related genes and signaling pathways and provided a comparative analysis of the DEGs from *R. philippinarum* hepatopancreas in response to *V. anguillarum* stimulation, with most genes activating the innate immune response (32, 33). These data laid the foundation for studying the innate immune systems and defense mechanisms in *R. philippinarum* (13). In the current study, RNA-seq was conducted to compare the immune responses of VaR, VaS, and Con clams to *V. anguillarum* infection. The results showed that some genes were highly expressed in the VaR group, which might be related to the resistance of *R. philippinarum* to *V. anguillarum*. These findings provide new insights for the immunological study of the response of *R. philippinarum* to *V. anguillarum*.

HSPs are generated in response to stress and have important roles in protein folding and the protection of proteins from denaturation or aggregation (34). Small HSPs (HSPs) associate with nuclei, cytoskeleton, and membranes and, as molecular chaperones, they bind partially denatured proteins, thereby preventing irreversible protein aggregation during stress. The upregulation of *Tg*sHSP after *V. parahaemolyticus* and lipopolysaccharide (LPS) challenge showed that sHSPs have a pivotal role in *Tegillarca graosa* antibacterial immunity (35). HSP70 gene expression in *M. galloprovincialis* hemocytes is triggered by *V. anguillarum*, but not by *V. splendidus* or *Micrococcus lysodeikticus* (36). Our results showed that the stress of *V. anguillarum* could lead to high expression of HSP70 gene (evm.TU.xfSc0003944.2) in different resistances and the difference folds were similar. Interestingly, in the transcriptome sequencing data, the expression of HSP70 (evm.TU.xfSc0000279.7) and HSP70 (evm.TU.xfSc0000353.1) genes in the resistant group was significantly higher than that in the susceptible and control groups. In this study, we only selected one HSP70 (evm.TU.xfSc0003944.2) gene for RT-qPCR verification, and the results were consistent with the sequencing results, indicating the relative accuracy of the sequencing data. The significant high expression of the other two HSP70 (evm.TU.xfSc0000279.7) and (evm.TU.xfSc0000353.1) genes in the resistance group can better explain their important role in the process of resistance to *V. anguillarum*. We predict that there are many members of the HSP70 gene family, and their specific expression is different. For the study of all members of the HSP70 family, as well as the study of the specific anti-*V. anguillarum* characteristics of some members, further research on the HSP70 gene family is required. Others studies have also shown challenge of Pacific abalone with heat shock or *V. anguillarum* dramatically increased the HSP70 mRNA expression level in muscle, followed by a recovery to normal levels after 96 h. By contrast, the HSP70 expression level in gills peaked at 12 h and was maintained at a relatively high level compared with the control after either thermal or bacterial challenge (37). Other studies reported increased expression of the HSP70 gene in most tissues after *V. anguillarum* infection (38, 39). The current results were similar, with the HSP70 gene showing a high expression level following infection with *V. anguillarum*. The difference in HSP70 gene expression in the VaR group compared with the Con group was higher than that of the VaS group (**Figure 8**). Therefore, overexpression of HSP70 gene might increase the ability of *R. philippinarum* to respond to *V. anguillarum* infection.

Complement component 1q (C1q), with a characteristic C1q globular domain, is an important pattern recognition molecule in the classical complement system and has a major role in crosslinking in some invertebrates (32, 40). According to the current transcriptome results, the c1q gene showed significantly increased expression in the VaR compared with the Con group, whereas it was decreased in the VaS group. The RNA-seq results were consistent, indicating that the c1q gene has an important role in the stress response to *V. anguillarum* (**Figure 8**).

GSTs are important enzymes involved in phase II detoxification and function by conjugating with the thiol group of glutathione; they can be used to study putative xenobiotic responses and to viral and bacterial infections (41). The transcription of GST increases in response to viral attack and other stressors (42). GST genes showed a high level of expression in *Exopalaemon carinicaudain* following infection with *V. anguillarum* (42). In *R. philippinarum*, the GST gene was highly expressed during stimulation by polycyclic aromatic hydrocarbons. These results demonstrated that benzo[a]pyrene significantly affected the expression of GSTr mRNA in the digestive gland of *R. philippinarum* and suggest that the GSTr gene has an important role in the biotransformation of benzo[a]pyrene (43). Interestingly, the current results showed that expression of GST genes in the VaR group was significantly lower than that in the Con and VaS groups. In *Pinctada martensii*, it was reported that a decrease in PmMGST3 mRNA abundance in hemocytes within the first 6 h after challenge was the result of the synergistic interaction of the immune and oxidative systems, hypotheses that could explain the temporal patterns of PmMGST3 expression seen after bacterial challenge (44). In the current study, the differential fold detection results for the GST gene expression in the VaR group were significantly lower than those in the Con and VaS groups. This is different from the GST gene expression of the above-mentioned species, such as *P. martensii*. The experimental detection time for *P. martensii* was 0–3 days, with a dynamic temporal expression pattern of the GST genes apparent during that time period. The transcriptome sequencing sampling time in this experiment was seven days for *V. anguillarum*. It is also possible that dynamic expression of the GST gene was present in the VaS and VaR groups during 1–6 days of the *V. anguillarum* challenge. Because of the temporal expression patterns of the GST genes in *P. martensii* during the immune process, we speculate that there is also a temporal pattern in *R. philippinarum*; thus, changes in the expression of the GST gene in *R. philippinarum* in response to *V. anguillarum* require further verification.

Lysozymes are found ubiquitously across the animal kingdom and have important roles in host immune responses against bacterial infection (45–48). Expression of the lysozyme gene in *R. philippinarum* was significantly upregulated after *V. anguillarum* infection, peaking at 48 h after infection, and then decreasing by 72 h, although the overall expression remained higher than that in the control group (48). In the current study, expression of the lysozyme gene in the VaR group was significantly lower than that in the Con group; thus, we speculate that there are temporal patterns in its expression similar to those seen for GST gene expression.

### Immunity effect of methyltransferase DEGs

The methyltransferase gene was highly expressed in *R. philippinarum* during infection with *V. anguillarum*. With research on the molecular responses of aquatic animals to pathogens, DNA methylation of the resulting immune effect has attracted increasing interest. In *Scophthalmus maximus*, the immune effect caused by methylation of the promoter regions of genes was reported (49). DNA methylation can occur under the action of DNA methyltransferase. DNA methyltransferase, a key enzyme mediating DNA methylation, is involved in numerous processes including genomic imprinting, X chromosome inactivation, transposable element suppression, and immune defense in vertebrate (50). DNA methylation leads to changes in DNA stability and the way in which DNA interacts with proteins without changing its nucleotide sequence (51). It has been reported that DNA methylation can cause some gene families to be up- or downregulated (52). In the current study, the methyltransferase gene was upregulated in the VaR and VaS groups and was significantly higher than that in the Con group. It was found that perch *Dicentrarchus labrax* larvae responded to *V. anguillarum* infection by increasing the expression of the methyltransferase gene (53). In a recent study, methyltransferases were significantly expressed in oysters subjected to LPS stress, demonstrating the important involvement of methyltransferases in the immune process (50). Some studies have also shown the adaptation of DNA methylation to the environment (54, 55). The current results suggest that there are resistance-related genes in the VaR group that were highly expressed during infection, which might enhance the immune function of *R. philippinarum*. However, further work is required to verify this.

**Figure 9:**
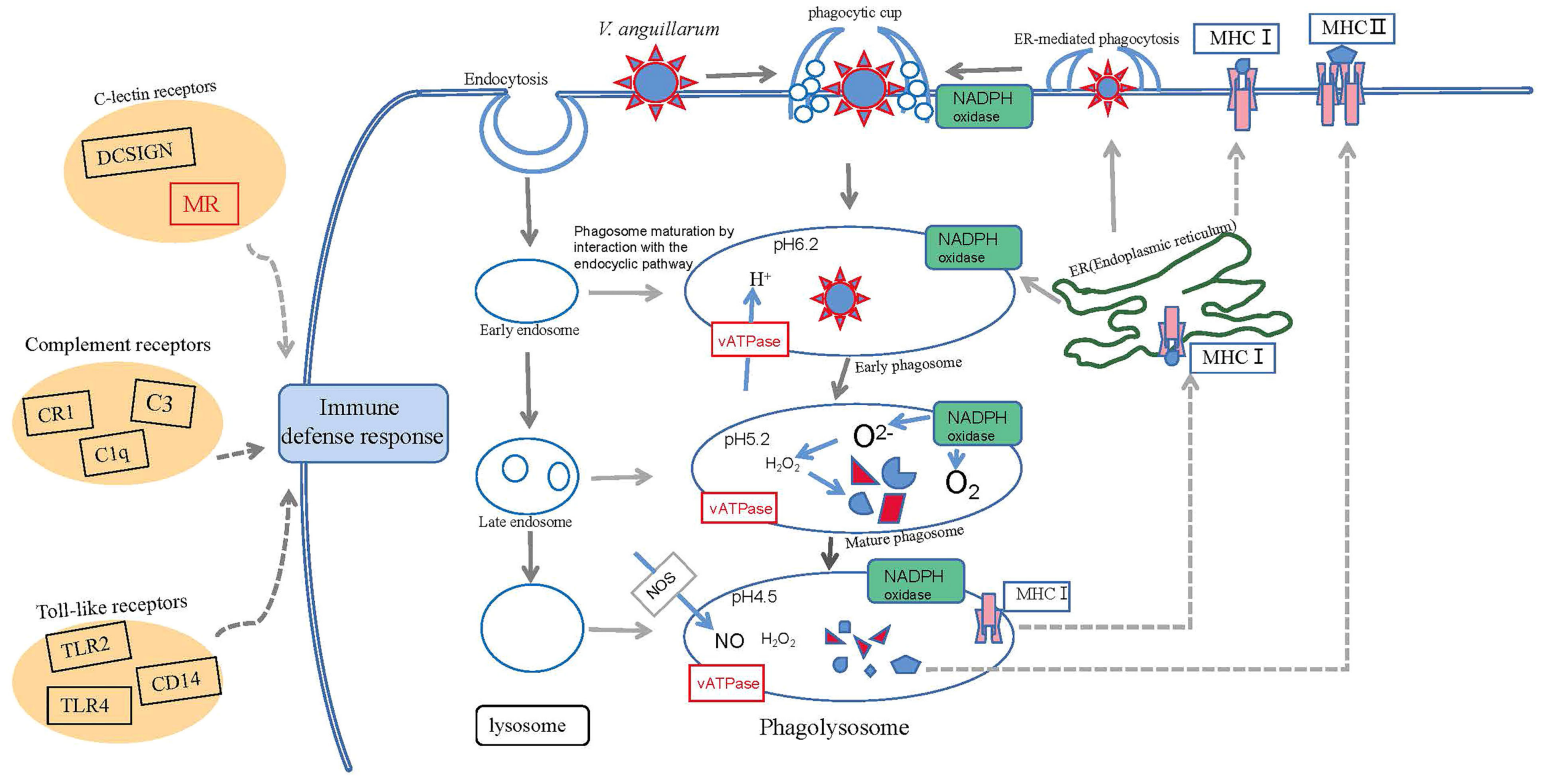
Conceptualization of mechanisms underlying immune defenses in *R. philippinarum* during challenge by *V. anguillarum*.

In the current study, we found some significantly upregulated pathways, such as those involving phagosomes. The phagosome (lgi04145) contained 19 differential genes in the VaR group, of which 12 were upregulated and seven were downregulated. The *TUBA* gene was significantly upregulated and, thus, we speculate that it is involved in the immune response to *V. anguillarum*. There were 24 DEGs in the VaS group, of which 16 were upregulated and eight were downregulated. Among them, those encoding F-actin and cathepsin were significantly upregulated. We speculate that these genes reduce the resistance of clams to *V. anguillarum.* Based on the results of the transcriptome analysis and the conclusions of previous studies, we have constructed the *R. philippinarum* phagosome pathways and the immune processes and immune pathways involved (**Figure 9**). When *V. anguillarum* is combined with cells, it is engulfed by the cell surface *via* endocytosis. The phagocytic process is divided into three phases, the early phagosome phase, the mature phagosome phase and the phagolysosome phase. Lysosomes are involved in the early endosome stage phagosome maturation by interacting with the endocyclic pathway. NADPH oxidase also has an important role in phagocytosis. NADPH not only has a central role in the reductive biosynthesis of cholesterol and fatty acids as well as elongation and desaturation of fatty acids, but also participates in the maintenance of cell integrity and detoxification (56). NADPH catalyzes the production of O_2_ and O^2–^ under the action of activating NADPH oxidase. Studies have shown that O^2–^ has an immune role through the formation of H_2_O_2_ (57). During phagocytosis, vATPase gene expression of resistant clams was significantly higher than that of the VaS group. The nitric oxide (NO) produced by NOS also has a role in this process. During phagocytosis, NO promotes fusion with lysosomes and exerts immune functions (58). According to the predicted results of KEGG, under the action of MHC genes, NO is transferred to the endoplasmic reticulum to resume the early phagocytic process, or transferred to the membrane surface for phagocytosis. MHC genes can be divided into MHCI and MHCII genes. In vertebrates their main function is to participate in adaptive immunity by specifically recognizing endogenous and exogenous antigens and presenting them to T cells (59–61). Due to the lack of adaptive immunity in mollusks, some PRRs such as C-lectin receptors, complement receptors, and TLR pathways play an innate immune role in the synergistic response of the entire phagocytosis process in the process of *R. philippinarum* resisting the infection of *V. anguillarum*. The C-type lectin (CTL) family has been extensively studied in both vertebrates and invertebrates (62). Invertebrate CTLs have been reported to have important roles in immune functions (63). CTL is one of the main receptors triggered in response to bacterial attack (5). In mollusks, several CTLs exhibited growth suppression activity against microbes (63). In the current study, sequencing the transcriptome revealed 340 C-type lectin gene. Two significant annotated genes were found to be involved in the phagosome pathway and both genes were upregulated after *V. anguillarum* infection. The expression level of the C-type lectin gene (evm.TU.xfSc0000193.14) in clams with resistance to *V. anguillarum* was significantly higher than that in the VaS clams. The Lectin C gene (evm.TU.xfSc0000495.7) in the VaS group was significantly higher than in the VaR and Con groups.

Interestingly, in the C-type lectin superfamily, we found that a class of MR genes also underwent significant changes in response to infection with *V. anguillarum*. MRs are PRRs that belong to type I transmembrane protein family (64). As a ‘nonstandard’ PRR, MR can bind to endogenous molecules and pathogens, mediate physiological clearance, and balance immune responses in the body to external stressors (65). Some studies have shown that there are two types of MR: MRC1 and MRC2. They have similar domains, such as fibronectin type II domains and multiple C-type lectin-like domains, but have different roles in cells. Compared with MRC1, which is involved in the innate immune response, MRC2 mediates the degradation of collagen in the lysosome (66). Recently, the corresponding full-length MR gene was cloned in species such as swimming crab and grass carp (67, 68). RNAi was used to silence the MR gene, which weakened the ability of the crayfish to eliminate bacteria. This indicates that MR is involved in antibacterial defense of crayfish (69). The immune importance of MR genes has been demonstrated in aquatic animals like grass carp, crayfish, and *Epinephelus coioides* (68–70), although little research has been done in *R. philippinarum.* Therefore, the function of the MR gene in the immune response of *R. philippinarum* to *V. anguillarum* requires further study.

Research has shown that the upregulation of HSP70 plays a critical role in modulating LPS-induced NF-κB activation and cytokine expression (71). The MyD88-independent pathway activates the transcription factor NF-κB and mitogen-activated protein kinases (MAPKs), which results in the production of induced inflammatory cytokines (71, 72). Many important members of the toll-like receptor signaling pathway dependent on the NF-κB pathway are differentially expressed in our results, such as TLR4, MyD88, TRAF6, IKK, NF-κB. Previous study also showed the involvement of TLR genes in *R. philippinarum* in resistance to *V. anguillarum* infection (16). Therefore, the toll-like receptor signaling pathway play important roles in response to *V. anguillarum* infection in *R. philippinarum.* In the current study, we preliminarily elucidated the molecular characteristics associated with the response of hepatopancreatic tissue from *R. philippinarum* to *V. anguillarum* challenge.

In addition to the above-validated genes, we also verified Ras, lectin, zf-C2H2, plasminogen, and F-box genes. Ras is an important superfamily of signaling proteins found in eukaryotes, its family members are indispensable in innate immunity, phagosome formation and maturation, and pathogen clearance (73). The plasminogen gene belongs to the C-type lectin (CTL) family, and CTLs are pattern recognition receptors (PRRs) that play important roles in the identification and elimination of pathogens by the innate immune system (18). The protein gene zf-C2H2 containing the BTB/POZ domain has been reported to be related to its growth and development in zebrafish (74). Our results showed that the expression of zf-C2H2 gene was significantly up-regulated in resistant individuals and down-regulated in susceptible individuals. In susceptible individuals, the expression of the zf-C2H2 gene was decreased. Therefore, we speculate that the high expression of zf-C2H2 gene may enhance the tolerance of *R. philippinarum* to *V. anguillarum*. F box-containing proteins are part of Skp1-Cullin-F box (SCF) E3 ubiquitin ligase complexes, which transfer ubiquitin from an E2 ubiquitin-conjugating enzyme to a target protein (75). Studies have shown that ubiquitination plays an important role in vertebrate immunity. Recent studies have shown that the ubiquitin-conjugating enzyme CgUbe2g1 is involved in the innate immune response of *Crassostrea gigas* against pathogenic microorganisms (76). The expression level of F-box gene detected by qPCR is not consistent with the RNA-seq results. This discrepancy may be due to a bias in the transcriptome sequencing or genotyping errors (77). Overall, the expression trends of the selected genes were largely consistent with the transcriptome analysis results.

Comparing the significantly differently expressed genes in the resistant clam group VaR and the nonresistant clam group VaS compared with the Con group in response to challenge with *V. anguillarum* has increased our understanding of the genes involved in the immune response of *R. philippinarum*. Such understanding of anti-*V. anguillarum*-related genes and response pathways provide references for future immunological research on *R. philippinarum* infected with *V. anguillarum*.

## Conclusions

Comparing the transcriptomes of *R. philippinarum* showing different responses to infection with *V. anguillarum* revealed gene expression differences between resistant and susceptible clams. Several immune-related genes, such as *HSP70*, *C1q*, *GST*, *lysozyme*, and *methyltransferase* were identified as critical genes for the resistance of Manila clam under *V. anguillarum* challenge. The C1q gene was significantly expressed in the resistant group and low in the susceptible group, proving the importance of the c1q gene in the process of resistance to *V. anguillarum* infection. In addition, some epigenetic-related methyltransferase gene showed significant changes, suggesting that epigenetic modification is integral to respond to this immune response. The phagosome pathway was significantly enriched and many related genes were involved in immune response to *V. anguillarum*, including the coordinated response of NADPH oxidase and NO, which both have important roles in immune response and defense. Our findings provide insight for understanding the immune system and defense response of *R. philippinarum* to pathogen invasion and for future immunological studies of *R. philippinarum*.

## Data Availability Statement

The datasets presented in this study can be found in online repositories. The raw sequences for R. Philippinarum can be found at https://www.ncbi.nlm.nih.gov/bioproject/PRJNA811359. The RNA-seq datasets are available in the NCBI Sequence Read Archive (SRA) with accession numbers SRR18177730, SRR18177729, and SRR18177728.

## Author Contributions

HN and XY conceived the study and revised the manuscript. ZY and KJ conducted the experiment. ZY analyzed the data and wrote the draft manuscript. All authors contributed to the article and approved the submitted version.

## Funding

This work was supported by the Chinese Ministry of Science and Technology through the National Key Research and Development Program of China (2018YFD0901400, 2019YFD0900704) and the China Agriculture Research System Project (CARS-49), and the Scientific Research funding from Liaoning Provincial Department of Education (LJKZ0701).

## Conflict of Interest

The authors declare that the research was conducted in the absence of any commercial or financial relationships that could be construed as a potential conflict of interest.

## Publisher’s Note

All claims expressed in this article are solely those of the authors and do not necessarily represent those of their affiliated organizations, or those of the publisher, the editors and the reviewers. Any product that may be evaluated in this article, or claim that may be made by its manufacturer, is not guaranteed or endorsed by the publisher.
