# Supplementary material for: Molecular Mechanisms Underlying Vibrio Tolerance in Ruditapes philippinarum Revealed by Comparative Transcriptome Profiling
Source: Front Immunol. 2022 May 9;13:879337. doi: 10.3389/fimmu.2022.879337 (PMC9125321; doi:10.3389/fimmu.2022.879337)
Supplement: Supplementary file 1 [file Table_1.docx]

Table S1 Primers used for qPCR in this study.

| Primer name | Gene ID | Sequence (5′-3′) |  |
| --- | --- | --- | --- |
| C1q | xfSc0001698.6 | F | AGCATGACCGACGCTGATGTAACC |
|  |  | R | ACCCACCGCCCTGTCTAAATCTGA |
| Hsp70 | xfSc0003944.2 | F | GACCAGGAAGAGAAATGCCAGC |
|  |  | R | CTTTTCCGAATCGTTGCCATAA |
| MET-fkbm | xfSc0000835.3 | F | GAGACGAAGAGGTTGGACAGGTTA |
|  |  | R | TGTATCCTTTCTTGGTCCATTCGT |
| Ras | xfSc0001526.12 | F | GGAATGTGGTAGAGATGGGCTTA |
|  |  | R | CGGTGTCAGGATAGGAAAGAGG |
| Lectin | xfSc0000193.14 | F | GGGTGTCCGATGAGCAGAAAAT |
|  |  | R | TTTCACCTCCCGTACCTCCTTT |
| Plasminogen | xfSc0000495.7 | F | ATATCAAGCACTGGCGATGGAAGC |
|  |  | R | CAAGCGGAGGAATAACTACTGGTGTG |
| zf-C2H2 | xfSc0000897.2 | F | TATCTCACCTCCCTGTCTACCCTC |
|  |  | R | ATTGAAGGGAGCAGGATCTAAGG |
| GST-N | xfSc0000179.2 | F | TAGTCATAACAGTTTCTTCGGTCG |
|  |  | R | GCACTGTCCTTTTTACTACCATTC |
| Lysozyme | Sc0000010.4 | F | TCAACCGACTTTGGCATATTTC |
|  |  | R | GCTTGCGTCATTGCTTATGTTAG |
| F-box | xfSc0001601.2 | F | GCAGCCACCCTTCATACCATACG |
|  |  | R | TCCAGTAACGCTCAAGTTCCAATCTAC |
| β-actin | AY889707.1 | F | CTCCCTTGAGAAGAGCTACGA |
|  |  | R | GATACCAGCAGATTCCATACCC |
