# Supplementary material for: Molecular Mechanisms Underlying Vibrio Tolerance in Ruditapes philippinarum Revealed by Comparative Transcriptome Profiling
Source: Front Immunol. 2022 May 9;13:879337. doi: 10.3389/fimmu.2022.879337 (PMC9125321; doi:10.3389/fimmu.2022.879337)
Supplement: Supplementary file 2 [file Table_2.docx]

Table S2. Summary of the number of deaths for each of the daily *V. anguillarum* challenge and control groups and the average cumulative mortality for the 3 parallel groups.

| Experiment days | *V.anguillarum* | Control |  | *V.anguillarum* | Control |
| --- | --- | --- | --- | --- | --- |
|  | number of deaths | |  | cumulative mortality rate (%) | |
| 1 | 8 | 2 |  | 2.7 | 0.7 |
| 2 | 10 | 0 |  | 6.0 | 0.7 |
| 3 | 10 | 0 |  | 9.3 | 0.7 |
| 4 | 14 | 0 |  | 14.0 | 0.7 |
| 5 | 10 | 0 |  | 17.3 | 0.7 |
| 6 | 17 | 1 |  | 23.0 | 1.0 |
| 7 | 39 | 1 |  | 36.0 | 1.3 |
| 8 | 24 | 1 |  | 44.0 | 1.7 |
| 9 | 15 | 1 |  | 49.0 | 2.0 |
| 10 | 9 | 1 |  | 52.0 | 2.3 |
| 11 | 9 | 0 |  | 55.0 | 2.3 |
| 12 | 9 | 0 |  | 58.0 | 2.3 |
| 13 | 7 | 0 |  | 60.3 | 2.3 |
| 14 | 7 | 0 |  | 62.7 | 2.3 |
| 15 | 2 | 0 |  | 63.3 | 2.3 |
| 16 | 0 | 0 |  | 63.3 | 2.3 |
| total | 190 | 7 |  |  |  |
